# Rise of monkeypox: Lessons from COVID-19 pandemic to mitigate global health crises

**DOI:** 10.1016/j.amsu.2022.104049

**Published:** 2022-06-22

**Authors:** Farah Ennab, Faisal A. Nawaz, Kapil Narain, Goodluck Nchasi, Mohammad Yasir Essar

**Affiliations:** College of Medicine, Mohammed Bin Rashid University of Medicine and Health Sciences, Dubai, United Arab Emirates; Nelson R. Mandela School of Medicine, University of KwaZulu-Natal, Durban, South Africa; The Catholic University of Health and Allied Sciences, Mwanza, Tanzania; Kabul University of Medical Sciences, Kabul, Afghanistan

## 1 Introduction

The emergence and spread of infectious diseases with pandemic potential has occurred regularly throughout history. HIV/AIDS, bubonic plague, smallpox, and influenza are some of the highest-profile examples. The COVID-19 outbreak was declared a pandemic on March 11, 2020, by the World Health Organization (WHO). Alongside other measures such as restrictions on population mixing and the use of face masks, COVID-19 vaccination has been arguably the most effective intervention in reducing deaths and severe COVID-19 disease. As of May 23, 2022, almost 12 billion vaccine doses have been administered globally [1].

Monkeypox is a rare viral zoonotic disease caused by the monkeypox virus. This virus has 2 strains, the West African and the Congo Basin clades [2]. On May 13, 2022, the WHO reported several cases of monkeypox virus infections, across three of its regions: namely Africa, the Americas, and the European Region. As of the June 8, 2022, about 1285 laboratory-confirmed cases were detected in 28 regions [3]. This rare viral disease commonly endemic in West and Central Africa has now been detected in more than 11 non-African countries such as the United Kingdom (UK) and the United States of America. Health authorities in the United Kingdom established a special task force to coordinate the extensive contact tracing of people who had contact with the confirmed cases. In addition, vaccination has been offered to higher-risk contacts [4].

The SARS-CoV-2 virus is typically spread by respiratory aerosols or droplets. Monkeypox can be transmitted via respiratory aerosols, but transmission can be via contact with lesions, bodily fluids, and contaminated materials such as bedding and towels. The basic reproduction number for monkeypox appears to be much lower than COVID-19, hence transmission should be slower than has been observed from COVID-19 outbreaks [5]. Some patients may display clinical features including the classical skin rashes and listers, along with symptoms such as headache, fever, swollen lymph nodes, muscle and body aches, back pain, and exhaustion. The West African strain is the type of monkeypox virus responsible for the 2022 outbreak, and this strain appears to cause less severe disease compared to the Congo Basin strain, with a case fatality rate of 3.6% compared to 10.6% for the Congo Basin strain [6]. The pandemic response to COVID-19 has provided significant new knowledge, some of which could be adapted and applied to the 2022 Monkeypox outbreak, minimizing its spread and reducing the global impact.

## 2 Emerging public fear and UANIDS concern of stigmatization

Stigma and discrimination are perpetuated with the Monkeypox outbreak. Many of the cases confirmed across May and June 2022 are linked to men who have sex with men (MSM) networks, a demographic that is already stigmatized, for example through the emergence of AIDS [7]. Transmission of Monkeypox disease remains primarily skin to skin and the possibility of sexual transmission is yet to be determined. The close contact required during sexual contact is likely to be a key factor for transmission – there is no current evidence that the virus is in itself a sexually-transmitted infection such as HIV or herpes simplex virus. Moreover, some of the claims are likely to be discriminatory, homophobic, unfounded, and blatantly infringing the human rights of the LGBTI community that has consistently faced abuse. These concerns prompted the United Nations and WHO to release statements, highlighting that these claims could lead to underreporting of cases if they were to be reinforced by the public [8]. Monkeypox virus can affect anyone and is not linked to a particular sexual orientation. There are various reasons for this increase – noting that some cases were observed from people who attended festivals, saunas, and parties, and the lack of social distance which is known to increase the likelihood of viral infections [9].

Additionally, whilst cases of Monkeypox disease have been reported in over 30 countries in Europe, the UK, Australia, Canada, and the Middle East, images of the typical skin presentation have sometimes been from Africa, incorrectly suggesting the outbreak as an African disease. This can be seen to be reinforcing discrimination and racism in global health and once again exposing the colonial mindsets of public commentators [10]. Such discrimination poses immense barriers to health equity, exacerbating the existing health challenges and access to treatment for these populations that are already overburdened with stigma. A holistic community response with leaders from various sectors is needed to ensure health for all. Encouragingly, some media articles have shown images of monkeypox blisters on different skin tones [11].

It is important that public health messaging highlights the individuals at highest risk, whilst remaining free of discrimination.

## 3 Lessons learned from the COVID-19 pandemic and a future strategy proposal

### 3.1 Early containment measures

Early containment measures taken during the COVID-19 pandemic on a national level have been beneficial in mitigating the spread of infection. This was particularly seen in China in 2020 under the “zero covid” policy which implied zero tolerance for local transmission. However, their continued attempts in 2022 to maintain zero COVID, amid a low uptake of vaccination, are widely criticized [12].

National practices include identifying infected cases earlier in the outbreak phase and offering isolation arrangements, close contact tracing and quarantine, and restrictions of travel from areas with community transmission. Sustained containment prevented a destructive second COVID-19 wave, with only three COVID-19-related deaths among 15 500 cases (case-fatality ratio <0.05%) from April 2020 through March 2021 in mainland China. New Zealand's national pandemic response is another example of effective containment through timely interventions. By creating a strategic policy for detection, isolation, and treatment of new cases, the time from onset of symptoms to notification had been reduced from 9·7 days (95% uncertainty interval 8·8 to 10·7) to 1·7 days (1·2 to 2·2), and the time from onset to isolation from 7·2 days (6·3 to 8·2) to −2·7 days (−4·7 to −0·8), meaning that people were isolating an average of 2·7 days before illness onset [13]. The lessons learned from such strategies also highlight areas of improvement for future outbreaks. Although the asymptomatic cases accounted for a small proportion of the disease burden in New Zealand, many asymptomatic individuals remained undetected despite targeted testing of groups less likely to show symptoms in the later phases. The Monkeypox outbreaks serve as an opportunity to improve the timely nature of preventing global catastrophes. Understanding the role of various stakeholders involved in implementing early-interventional strategies is key to combatting the social, financial, and health-related implications of underprepared response systems.

### 3.2 Enhancing public trust through social media and technology

The implementation of digital measures for promoting public health awareness during the COVID-19 pandemic has fostered a double-edged sword for health-related information. While misinformation has been a constant struggle on a global scale, there have been positive aspects of using social media as an accessible tool for knowledge-sharing during this period. The United Arab Emirates (UAE) government had launched the *#TogetherWeRecover* social media campaign, which has become one of the most impactful initiatives in the country [14]. As part of the campaign, the UAE National Emergency Crisis and Disaster Management Authority (NCEMA) posted various messages on social media platforms and public spaces to encourage vaccinations and practice safety precautions amongst UAE residents. By engaging with the hashtag campaign, the citizens and residents were well-informed and active in showing their support for the national effort to counter this national challenge [15]. Dissemination of vital information using televised news outlets, broadcasting, and radio announcements in several languages, including English, Arabic, Urdu, Filipino, and Mandarin, thereby reflecting the need for focusing on the diversity of regional populations. Along with government efforts to engage the public for precautionary measures, the use of digital health technologies in disease surveillance and control has played an instrumental role during the COVID-19 pandemic. This includes role models of digital health innovations in lower-income countries. Drones were utilized to share health awareness messages in Rwanda, and for disinfecting public areas in Ghana [16,17]. Contactless soap dispensers and solar-powered hand washing sinks were used to encourage positive hygiene in Senegal and Uganda. Telehealth services have surged to adapt with the growing demands of the COVID-19 pandemic. This symbiotic overlap of technology with healthcare allows for devising efficient triage systems amidst the rising cases of Monkeypox outbreaks worldwide [18,19].

### 3.3 Early rollout of therapeutic medical therapies and global robust research

During the early stages of the COVID-19 pandemic, there was a notable absence of concrete and efficacious treatment regimens put in place by healthcare policymakers or recommended by existing international guidelines. As a result of this and in an attempt to prevent further disease progression at an early point of the pandemic, clinicians worldwide quickly started utilizing various medical therapeutic interventions. These measures included the usage of the anti-viral agent remdesivir which was proven to reduce recovery time in patients hospitalized with COVID-19 after being rolled out as part of an ongoing clinical trial at the time known as the adaptive COVID-19 treatment trial (ACTT), a randomized placebo-controlled trial [20]. In addition, another clinical trial known as the “RECOVERY” trial which was funded by the medical research council and the national institute for health research had evaluated the efficacy of dexamethasone and ultimately proved that dexamethasone reduced mortality rates in patients hospitalized with COVID-19 and those who required mechanical ventilation or high-flow oxygen [21]. Due to the rigorous efforts of these trials, both of these medications were successfully approved for the treatment of COVID-19 on a global scale and were later introduced in different treatment guidelines. Furthermore, there were few other medical interventions that were widely used to treat this infection, such as anti-malarial agents, antibiotics, and oxygen therapies [22,23]. Although these measures did not have such strong evidence to support their efficacy in reducing morbidity and mortality rates, they allowed for perpetual and robust efforts by researchers globally to move forward with advancements in pharmaceutical treatments ranging from conducting robust clinical trials to drug discovery. These efforts, coupled with global support from volunteers and scientists resulted in a favorable health outcome for many patients affected by COVID-19 [24]. Hence, we anticipate a similarly positive result to the current situation of the Monkeypox virus if emphases on therapeutic interventions as well as continuing research efforts were to be recognized and implemented early.

### 3.4 Multiple vaccination manufacturing efforts and vaccine hesitancy campaigns

Throughout the history of known global pandemics, the dire need to develop and manufacture therapeutic vaccines has always been at the forefront of public health policymakers. The profound impact of COVID-19 had drawn remarkable attention from scientists to accelerate the process of vaccine development [25]. Within a strikingly short period of time, multiple vaccines were available to the public. As of the January 12, 2022, an example of these available vaccines included: The Sinopharm vaccine, Pfizer/BioNTech, AstraZeneca/AZD1222, Moderna (mRNA1273), and Janssen/Ad26.COV 2.S vaccine developed by Johnson & Johnson [26]. To date, approximately 11.83 billion doses have been administered worldwide with around 65.8% of the world population having received at least a single dose of a COVID-19 vaccine [27]. It has been widely proven that the available COVID-19 vaccines effectively reduce the mortality rate, decrease the hospitalization rate, and prevent severe disease manifestation [28]. These notable statistics serve as a testimony to the global effort that was put in the early stages of the pandemic in the manufacturing, development, and distribution strategies for a potential COVID-19 vaccine. These efforts, in addition to how the genomic sequence or genomic blueprint of this virus was shared online within counted days, granted the world's scientists a significant advantage with quick and efficient research work to take place at the early stages of the pandemic [29]. Furthermore, various educational campaigns and community sessions to combat vaccine hesitancy were established through a successful loop of communication between medical personnel and the general public [30]. This increased community awareness played a crucial role in disseminating the vaccines and increasing vaccine uptake. It is due to these early yet extremely effective vaccine measures that the COVID-19 pandemic is being currently contained at an impressive speed. We strongly encourage community-oriented interventions in the battle against the Monkeypox virus, hoping to draw from this successful model of vaccine development strategies and global rollout.

## 4 Conclusion

Monkeypox is a viral zoonotic disease with clinical manifestations which are similar to smallpox but are less severe, is now re-emerging in an unprecedented outbreak, and is concurrently being reported widely in non-endemic areas. As the world is still recovering from the COVID-19 pandemic's repercussions, this viral outbreak poses a significant threat to global public health. The limited available data we have about Monkeypox may underestimate the severity of this infection and could lead to an exacerbated global reach of cases. By paying close attention to the lessons learned from the COVID-19 pandemic ranging from early strategic containment measures, utilization of community-oriented public awareness, transparency of scientific evidence sharing, and global robust research efforts put into vaccine development and therapeutic interventions, a potential global health catastrophe can be avoided.

## Ethical approval

N/A.

## Sources of funding for your research

None.

## Author contributions

All individuals who meet authorship criteria are listed as co-authors who have participated adequately in this work to take public responsibility for the generated results and content, including participation in the idea, design, writing of the manuscript, or revision. Furthermore, each author listed certifies that this material or similar material has not been and will not be submitted to or published in any other publication before its appearance in **The Annals of Medicine and Surgery.**

Conceptualization: FE, FN, ME; Writing original draft: FE, FN, GN, KN; Review and editing: FE, FN, KN, ME.

## Registration of research studies

1. Name of the registry: N/A.
2. Unique Identifying number or registration ID: N/A.
3. Hyperlink to your specific registration (must be publicly accessible and will be checked): N/A

## Guarantor

N/A.

## Consent

N/A.

## Declaration of competing interest

None declared.
